# Notch1 Is Pan-Endothelial at the Onset of Flow and Regulated by Flow

**DOI:** 10.1371/journal.pone.0122622

**Published:** 2015-04-01

**Authors:** Espen D. Jahnsen, Alexandre Trindade, Hans C. Zaun, Stéphanie Lehoux, António Duarte, Elizabeth A. V. Jones

**Affiliations:** 1 Lady Davis Institute for Medical Research, McGill University, 3755 Côte Ste Catherine, Montreal, Quebec, H3T 1E2, Canada; 2 Department of Biomedical Engineering, McGill University, 3775 University St, Montreal, QC, H3A 2B4, Canada; 3 Centro Interdisciplinar de Investigação em Sanidade Animal, Faculdade de Medicina Veterinária, University of Lisbon, Lisboa, Portugal; 4 Instituto Gulbenkian de Ciência, Oeiras, Portugal; 5 Department of Cardiovascular Science, KU Leuven, UZ Herestraat 49—box 911, 3000, Leuven, Belgium; Feinberg Cardiovascular Research Institute, Northwestern University, UNITED STATES

## Abstract

Arteriovenous differentiation is a key event during vascular development and hemodynamic forces play an important role. Arteriovenous gene expression is present before the onset of flow, however it remains plastic and flow can alter arteriovenous identity. Notch signaling is especially important in the genetic determination of arteriovenous identity. Nevertheless, the effect of the onset of circulation on Notch expression and signaling has not been studied. The aim of this study is therefore to investigate the interaction of Notch1 signaling and hemodynamic forces during early vascular development. We find that the onset of *Notch1* expression coincides with the onset of flow, and that expression is pan-endothelial at the onset of circulation in mouse embryos and only becomes arterial-specific after remodeling has occurred. When we ablate flow in the early embryo, endothelial cells fail to express Notch1. We show that low and disturbed flow patterns upregulate *Notch1* expression in endothelial cells *in vitro*, but that higher shear stress levels do not (≥10 dynes/cm^2^). Using siRNA, we knocked down Notch1 to investigate the role of Notch1 in mechanotransduction. When we applied shear stress levels similar to those found in embryonic arteries, we found an upregulation of *Klf2*, *Dll1*, *Dll4*, *Jag1*, *Hey1*, *Nrp1 and CoupTFII* but that only *Dll4*, *Hey1*, *Nrp1* and *EphB4* required Notch1 for flow-induced expression. Our results therefore indicate that Notch1 can modulate mechanotransduction but is not a critical mediator of the process since many genes mechanotransduce normally in the absence of Notch1, including genes involved in arteriovenous differentiation.

## Introduction

Blood flow is an important biological regulator and initiates and maintains many events during embryonic development. Shear stress, a mechanical force created by blood flow, is an important factor regulating many physiological functions. Although there is expression of arterial and venous specific genes in the vasculature before the onset of flow [1, 2], there are no structural differences between the vessels [3]. With the entry of erythroblasts into circulation, morphologically distinct arteries and veins develop through the process of vascular remodeling [4]. Though arterial- and venous-specific genes are present before this occurs, altering the flow can change an artery into vein and vice versa, both in terms of morphology and gene expression [5, 6], indicating plasticity in vessel identity.

Genetic predetermination of arterial identity has been shown to occur through the Notch signaling pathway. Notch receptors and ligands are involved in a plethora of developmental processes including somite coordination [7, 8], cardiovascular formation [9], and neuronal development [10]. Mutations in the Notch pathway cause pathologies such as Alagille Syndrome [11, 12], cerebral autosomal dominant arteriopathy with subcortical infarcts and leukoencephelopathy (CADASIL, [13]) and Tetralogy of Fallot [14]. In zebrafish, Sonic Hedgehog expressed by the notochord induces expression of vascular endothelial growth factor (VEGF) in the somites, which upon secretion creates a diffusion gradient [15, 16]. High levels of VEGF upregulate *Notch* and *Dll4* expression and initiate arterial differentiation. In regions of low VEGF concentrations, however, venous identity prevails [15, 16]. In mouse, such a clear picture of the genetic pathway for arterial differentiation has not yet been established. Within the Notch pathway, only *Dll4* and *Hey1* are expressed in an arterial specific manner before the onset of flow [17]. Dll4 is a Notch ligand, however, and it is not clear how it is signaling at this stage in the absence of any Notch receptors. Hey1 is a transcription factor whose expression is induced by Notch activation. Therefore, in the mouse, arterial differentiation is present before the onset of blood flow but the majority of the Notch signaling pathway is not expressed until after flow begins.

In this work, we have investigated the expression of *Notch1* just after the onset of circulation in mouse embryos. We found *Notch1* expression begins with the onset of flow but is pan-endothelial at the stage where erythroblasts enter circulation. Only by E9.5, after vascular remodeling has occurred, is *Notch1* restricted to arteries. Using a technique we previously developed [18], we ablated flow in developing mouse embryos and cultured them for 24 hours. We find in the control embryos, that *Notch1* expression was arterial after culture while no expression in either arteries or veins was observed in the embryos with ablated flow, indicating that *Notch1* requires flow to be expressed. We investigated the patterns of flow that could regulate *Notch1* expression and found the largest increase occurred at low levels of laminar shear stress (1–5 dynes/cm^2^) and with oscillatory flow types (either 0 ± 3 or 2 ± 3 dynes/cm^2^). Not only could *Notch1* expression be upregulated by flow, but the expression of Notch ligands and transcription factors were also induced by exposure to shear stress. We therefore knocked down Notch1 and investigated the effect on flow-induced expression of Notch signaling molecules as well as typical shear-induced genes and downstream targets in arterial-venous differentiation. Flow could induce the expression of approximately half the genes investigated, even in the absence of Notch1. Overall, our results indicate that *Notch1* expression occurs after the onset of flow and does not become arterial-specific until remodeling has occurred. While Notch1 is required for the mechanotransduction of some genes, our results indicate that it is not an essential component of mechanotransduction since the flow-induced expression of many genes is not affected by Notch1 knockdown.

## Materials and Methods

### Whole-mount in situ hybridization and flow ablation in embryos

All procedures were approved by the Animal Care Committees of McGill University and we have followed recommendations of the Canadian Council on Animal Care. *In situ* hybridization has been described previously [6] with probes for *Notch1* [19], *Notch4* [20], and *Hey1* [21]. Antibodies against digoxygenin Fab-fragments conjugated with alkaline phosphatase were purchased from Roche Diagnostics. Stained embryos were imaged and then equilibrated in 30% sucrose/PBS, embedded in tissue freezing medium and cryogenically sectioned. For flow ablation, embryos at 4 somites were dissected and flow was ablated using a previously published technique [18]. Briefly, the inlets to the heart were pinched off on both sides using #55 watchmaker forceps and embryos were cultured for 24 hours. After culture, embryos were verified to ensure that the yolk sac had inflated and a heartbeat was present, but no circulation or vascular remodeling was observed.

### Cells and cell cultures

Human abdominal aortic endothelial cells (HAAEC; Coriell Institute) were propagated through passage 5 in complete endothelial cell growth medium MV (PromoCell) supplemented with 1% Penicillin Streptomycin. Cells were expanded on 0.1% gelatin. For flow experiments, HAAEC were seeded at a density of 150 000 cells/mL on a culture slide, coated with 4% rat tail collagen type 1 and grown to confluence (3 days).

### In vitro flow apparatus

A parallel plate flow chamber was designed in-house. The parallel plate flow chamber was connected to a closed-loop perfusion system consisting of a vented media reservoir, a flow dampener, a peristaltic pump and/or a computer-driven syringe pump. The viscosity of the endothelial cell media was measured using a Bohlin CVO 120 HRNF Viscometer (Malvern Instruments) to be 1.02334 cP. Laminar flow was verified by seeding the perfusate with fluorescent microparticles and imaging with a high-speed camera on a fluorescent microscope. HAAEC were exposed to laminar flow at a calculated wall shear stress of 1 to 15 dynes/cm^2^ and oscillatory shear stress of 0 to 5 ± 3 dynes/cm^2^ as indicated. Static slides were cultured in parallel. After 1 hour, total RNA or protein was isolated.

### Quantitative PCR

Total RNA was extracted using an RNeasy Micro Kit (Qiagen). RNA concentration and purity was quantified on a NanoDrop. 1 μg of RNA was reverse transcribed using Oligo-d(T)_16_ primers (Applied Biosystems), and M-MLV reverse transcriptase (Invitrogen). Gene expression was analyzed on an ABI Prism 7900HT Sequence Detector with SA Biosciences RT^2^ Real-Time SYBR Green master mix (Qiagen). Quantitect Primer Assays (Qiagen) were used for *HPRT* (QT00059066), *18S* (QT00248682), *Dll1* (QT00057631), *Nrp1* (QT00023009), *VEGFR2* (QT00069818) and *Notch1* (QT01005109). All other primers were designed in-house (Table 1). The data was normalized to two endogenous controls, *HPRT* and *18S*, using the ΔΔCt method.

**Table 1:** Primers for quantitative PCR.

Gene	Forward primers	Reverse primers
*Cx40*	TAGGCAAGGTCTGGCTCACT	TGATCTGCAGCACCCAGTAG
*CoupTFII*	TGGTTCCAAACCAGTTTATTCTGTG	AAGTGCGTTTCCATCACATTGAG
*Dll4*	CAGTGGGCAGCGAAGCTACA	ACAGGCAGTGGTAGCCATCCTC
*EphB4*	GATGCCTGGAGTTACGGGATTG	TCCAGCATGAGCTGGTGGAG
*EphrinB2*	CTCCTCAACTGTGCCAAACCA	GGTTATCCAGGCCCTCCAAA
*Hey1*	CTGAGCAAAGCGTTGACA	TCCACCAACACTCCAAA
*Hey2*	GAACAATTACTCGGGGCA	TCAAAAGCAGTTGGCACATG
*Jag1*	AGGCCGTTGCTGACTTAG	GCAGAAGTGGGAGCTCAA
*Klf2*	CCTCCCAAACTGTGACTGGT	ACTCGTCAAGGAGGATCGTG
*Notch4*	GCCGATAAAGATGCCCAGGA	ATCCCAGTGGTTACGTTGGTGAG
*Nrp2*	ATACCACACCAAGGCTGGAG	ACCACCTAGTCCGGGAGAGT
*Vinculin*	CTTTGCTGCTACAGGGGAAG	GGATATGGGACGGGAAGTTT

### siRNA transfection

*Notch1* siRNA (SI00119035) and AllStars Negative Control siRNA (1027280) were obtained from Qiagen. 10nM siRNA diluted in 100μl OptiMem serum-free medium (Invitrogen) with HiPerfect Transfection Reagent (Qiagen) was added to 150 000 cells pre-seeded on a collagen-coated culture slide in 1mL of OptiMem without Penicillin Streptomycin. After 1h of transfection, 1.3mL complete endothelial cell growth medium was added to each slide and incubated for 48 hours. Transfection media was then replaced with 2mL of complete endothelial cell media and the samples incubated for another 24 hours before performing experiments. qPCR and Western Blot was used to measure knockdown. For western blot, primary antibodies for Notch1 were obtained from EMD Millipore (1:500, 04–1046), glyceraldehyde phosphate dehydrogenase (GAPDH) from Santa Cruz Biotechnologies (1:400, sc-32233).

### Statistical analysis

Data are presented as the mean ± standard error of the mean (SEM). Results were analyzed using ANOVA with Tukey’s post hoc test.

## Results

### Notch1 expression pattern requires blood flow and is pan-endothelial when flow begins, becoming arterial-specific by E9.5

**Fig 1:**
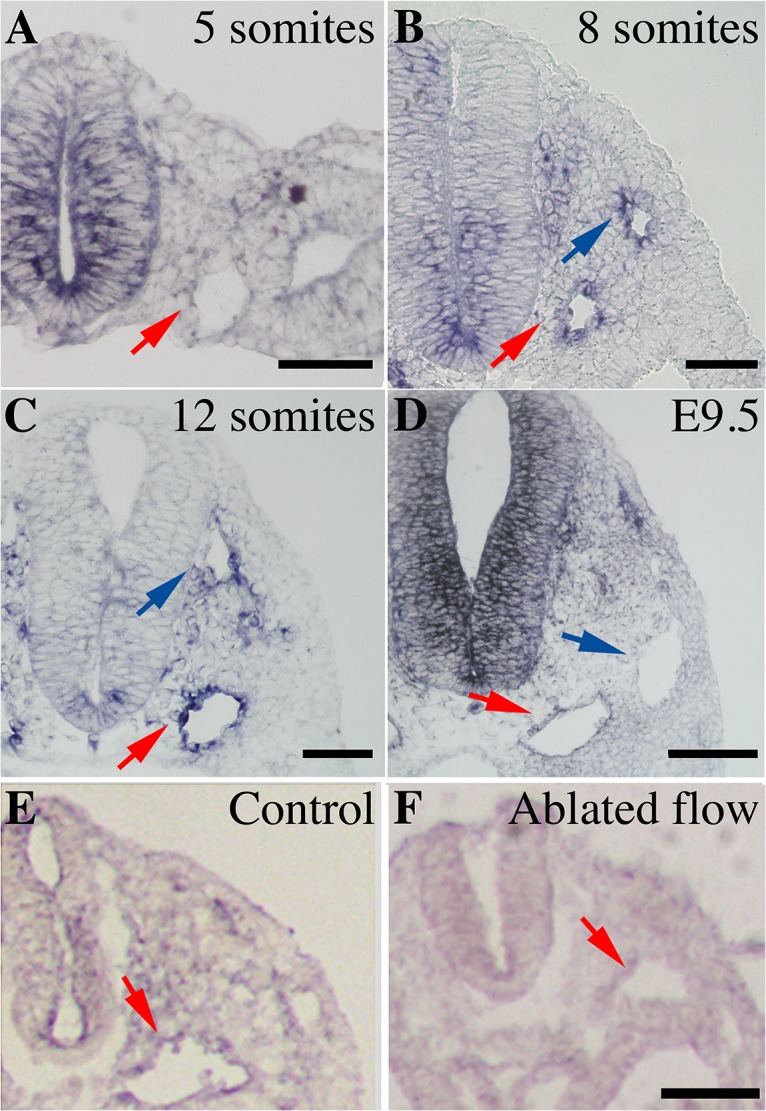
*Notch1* is pan-endothelial before vascular remodeling and only limited to the arteries after vascular remodeling. Expression of *Notch1* was studied by *in situ* hybridization on mouse embryos at 5 somites (A), 8 somites (B), 12 somites (C) and E9.5 (D, n = 6 per stage). *Notch1* expression is absent in arteries at 5 somites (red arrow). When blood flow initiates at 8 somites, *Notch1* expression is found both in arteries (red arrow) and veins (blue arrow) at 8 somites and at 12 somites. Venous expression of *Notch1* is absent after vascular remodeling at E9.5. To investigate if flow is required for the expression of Notch1, we stained for *Notch1* expression in embryos in which had ablated flow. *Notch1* expression is present in the dorsal aorta of the control embryos (E, n = 4) but no expression is observed in embryos with ablated flow (F, n = 5). Scale bar; 50μm (A-C), 100 μm (D-F).

Since Notch signaling has been implicated in the determination of arterial endothelial identity [15], we chose to look at the expression during early vascular development. In zebrafish, overexpression of the Notch1 intracellular domain is sufficient to induce arterial identity in veins [16]. We find *Notch1* expression is absent at 5 somites, before the onset of blood flow (Fig 1A, red arrow). Blood flow begins at E8.5 (between 6 and 8 somites, [4, 22]). At 8 somites, Notch1 is expressed both in the dorsal aortae (Fig 1B, red arrows) and in the anterior cardinal veins (Fig 1B, blue arrow). The expression is unchanged at 12 somites (Fig 1C), which represents a mid-remodeling stage. Once remodeling has occurred (E9.5), we find that *Notch1* expression becomes arterial specific (Fig 1D, red versus blue arrows). The presence of “arterial” gene expression in veins at 8 somites has previously been reported [17], though which arterial genes were expressed in the veins was not specified.

Since the onset of Notch1 expression coincided with the onset of blood flow, we investigated whether blood flow was required for *Notch1* expression. Using a technique that we previously published, we ablated flow in the embryos [18] and cultured the embryos for 24 hours. In control embryos, we find that *Notch1* expression is arterial after 24 hours of culture (equivalent to E9.25, Fig 1E, red arrow). In embryos lacking flow, we find no expression of *Notch1* in the arteries (Fig 1F, red arrow) or in the veins, indicating that *Notch1* requires blood flow for expression in the mouse embryo.

**Fig 2:**
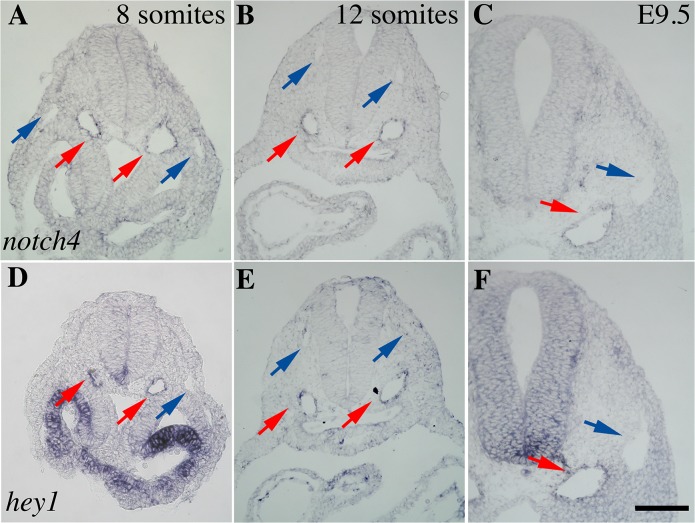
*Notch4* and *Hey1* are arterial specific at stages where Notch1 is pan-endothelial. *Notch4* (A-C) and *Hey1* (D-F) expression was verified by *in situ* hybridization on mouse embryos at 8 somites (A, D), 12 somites (B, E) and E9.5 (C, F, n = 6 per stage). At all stages investigated, *Notch4* (A-C) and *Hey1* expression was found to be arterial-specific (red arrows for arteries and blue for veins).

*Notch4* and *Hey1* have also been characterized as arterial-specific genes [17, 23]. Hey1^-/-^ embryos are viable, however the double knockout of Hey1 and Hey2 has a phenotype similar to Notch1 ablation [24]. Notch4 was also found to be non-essential for early vascular development but loss of both Notch1 and Notch4 results in a more severe phenotype than Notch1^-/-^ [16]. We performed *in situ* hybridization for *Notch4* (Fig 2A–2C) and *Hey1* (Fig 2D–2F) at the 8- and 12-somite stage and at E9.5. The expression of *Hey1* and *Notch4* is already restricted to the dorsal aortae at the 8-somite stage (Fig 2A–2D, red versus blue arrows). The same expression pattern is found at 12 somites (Fig 2B–2E) at E9.5 (Fig 2C–2F).

### Notch1 is upregulated by low shear stress and oscillatory shear stress in endothelial cells

**Fig 3:**
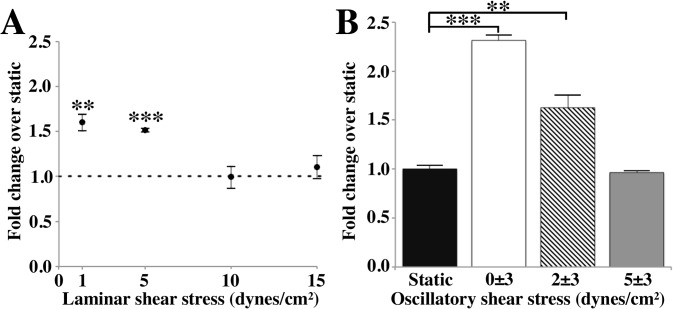
Low levels of laminar and oscillatory shear stress upregulate *Notch1* in endothelial cells. Human abdominal aortic endothelial cells were exposed to laminar shear stress between 1 and 15 dynes/cm^2^ and to three types of oscillatory flow for 1 hour. *Notch1* is upregulated by low levels of laminar shear stress (1–5 dynes/cm^2^, n = 4–10) but not by shear stress levels exceeding 10 dynes/cm^2^ (n = 4)(A). Oscillatory shear stress (0 ± 3 dynes/cm^2^, n = 4) and shear stress with some retrograde flow (2 ± 3 dynes/cm^2^, n = 16) also increase mRNA expression of *Notch1*, but pulsatile shear stress (5 ± 3 dynes/cm^2^, n = 4) does not (B). Expression was normalized to static control in all cases. All values are mean ± SEM., * p < 0.05; ** p < 0.01; *** p < 0.001; two-tailed Student’s t-test.

Since our results indicated that flow was necessary for the expression of *Notch1* in the embryo, we investigated the effect of different flow patterns on *Notch1* expression *in vitro*. Between 5 and 8 somites, erythroblasts enter circulation leading to an increase in shear stress [4, 22, 25]. The earliest blood flow has a retrograde component [26], becoming unidirectional by E9.5 [26]. The average magnitude of shear stress when flow initiates is between 2 and 5 dynes/cm^2^ in mouse embryos [27]. Differences in shear stress levels between embryonic arteries and veins have not yet been measured. However, it is known that the velocity of blood flow is about 1.5 to 2 times higher in embryonic arteries as compared to veins at stages between 6 and 12 somites [3]. As opposed to more mature vascular networks, strong pulsatility is present in both arteries and veins [3]. Human abdominal aortic endothelial cells (HAAECs) cultured on microscope slides were exposed to a range of steady laminar shear stresses between 1 and 15 dynes/cm^2^ for one hour. We find that low levels of laminar flow induce *Notch1* expression but that with higher levels of shear stress (≥10 dynes/cm^2^), expression is the same as static control (Fig 3A). Since significant retrograde flow is present in the embryo when erythroblast circulation begins, we also investigated the effect of flow reversal. We exposed endothelial cells to three types of pulsatility; fully oscillatory flow (0 ± 3 dynes/cm^2^) in which a complete reversal of flow direction is present; oscillatory flow with a slight reversal present (2 ± 3 dynes/cm^2^) and purely pulsatile flow with no retrograde component (5 ± 3 dynes/cm^2^). We find that both flow types in which flow reversal is present (0 ± 3 and 2 ± 3 dynes/cm^2^) induce an upregulation of *Notch1*, but that pulsatility without flow reversal does not (Fig 3B).

### Notch1 knockdown diminishes but does not ablate mechanotransduced gene expression

**Fig 4:**
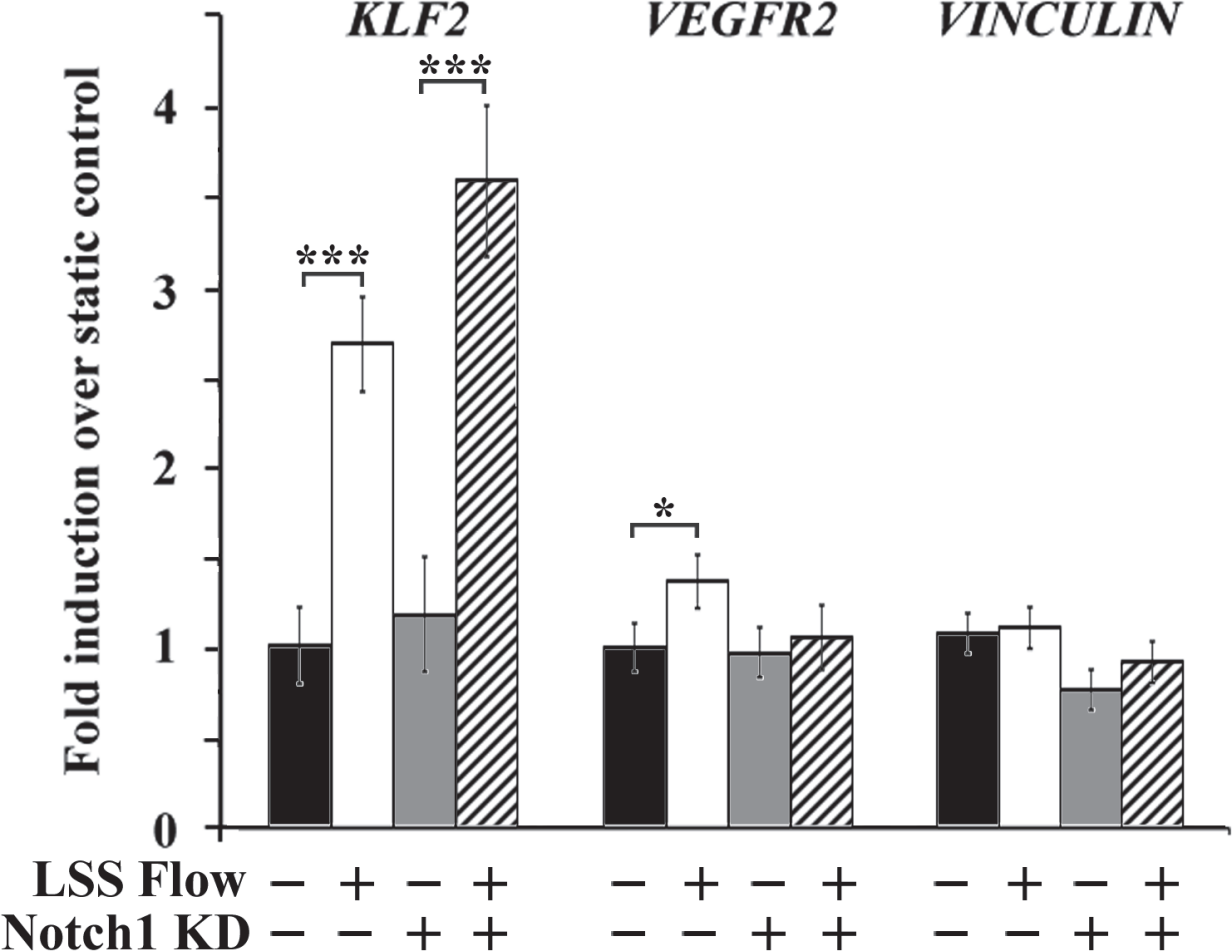
Notch1 knockdown does not affect shear-induced upregulation of *Klf2*. Cells with or without Notch1 were exposed to 5 dynes/cm^2^ of laminar shear stress for one hour. The expression of typical shear-induced genes was assessed. *Klf2* and *VEGFR2* were upregulated by low levels of laminar shear stress, but not *Vinculin*. Notch1 knockdown did not affect *Klf2* expression in response to flow, whereas it prevented the shear-dependent upregulation of *VEGFR2*. All values are mean ± SEM (n = 4–16 for all conditions). * p < 0.05; *** p < 0.001; Two-way ANOVA and Tukey’s post hoc comparisons.

The role of Notch1 in mechanotransduction has been studied surprisingly little. Shear stress induces Notch cleavage in embryonic stem cell-derived VEGFR2^+^ cells [28]. In zebrafish, ablating flow induces an increase in Notch signaling [29]. Other than that, a possible role for Notch in mechanotransduction or regulation of Notch signaling by shear stress has not been studied. We therefore investigated whether *Notch1* expression was required for mechanotransduction of typical shear-induced genes (*Klf2*, *VEGFR2*, *Vinculin*, Fig 4). We used siRNA transfection to knock down Notch1 in HAAECs. We achieved a 70% knockdown of Notch1 with or without flow (n = 15, SEM = 0.03). Effective knockdown was verified both by mRNA and protein expression. We used relatively low levels of shear stress (5 dynes/cm^2^), which corresponds to the average level of shear stress in the embryo at the onset of circulation but is also a level of shear stress in which we observed increased *Notch1* expression. Endothelial cells were exposed to flow for one hour. In the presence of control siRNA, exposing endothelial cells to laminar shear stress led to an upregulation of the genes *Klf2* and *VEGFR2*. The knockdown of Notch1 alone had no effect on the expression of these genes in static conditions, nor did it prevent the shear stress-dependent increase in *Klf2* expression. However, shear stress failed to upregulate VEGFR2 by shear stress when Notch1 was knocked down.

**Fig 5:**
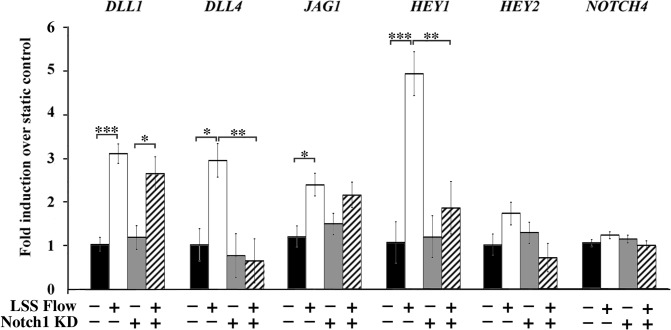
Notch1 is required for flow-induced regulation of half of Notch ligands and effectors. The expression of Notch ligands and effectors in response to flow (5 dynes/cm^2^ for one hour) was assessed. Except for *Hey2* and *Notch4*, all genes investigated were upregulated by laminar shear stress (5 dynes/cm^2^). When Notch1 was knocked down in cells, flow-induced upregulation of Notch ligands and effectors was significantly impaired, with the exception of *Dll1*. All values are mean ± SEM (n = 4–16 for all conditions). * p < 0.05; ** p < 0.01; *** p < 0.001; Two-way ANOVA and Tukey’s post hoc comparisons.

Masumura et al. reported that Notch intracellular domain (NICD) is cleaved by shear stress [28]. NICD induces the expression of transcription factors Hey1 and Hey2 [21]. Furthermore, Notch activation induces an amplification loop such that Notch activation induces expression of Notch ligands Dll4 and Jag1 [30, 31]. We therefore investigated the regulation of Notch targets by shear stress. We find that low levels of laminar shear stress alone upregulate most of the Notch ligands and effectors that we investigated (Fig 5), with the exception of Hey2. Knockdown of Notch1 alone does not affect the baseline expression of any of the genes investigated, but it does prevent the upregulation of Notch target genes, with the exception of *Dll1*, in response to flow. A positive feedback loop for amplification of *Dll1* by Notch signaling has not been shown unlike the other ligands tested. We also investigated whether Notch4 would compensate for the loss of Notch1. No changes in *Notch4* expression are observed.

**Fig 6:**
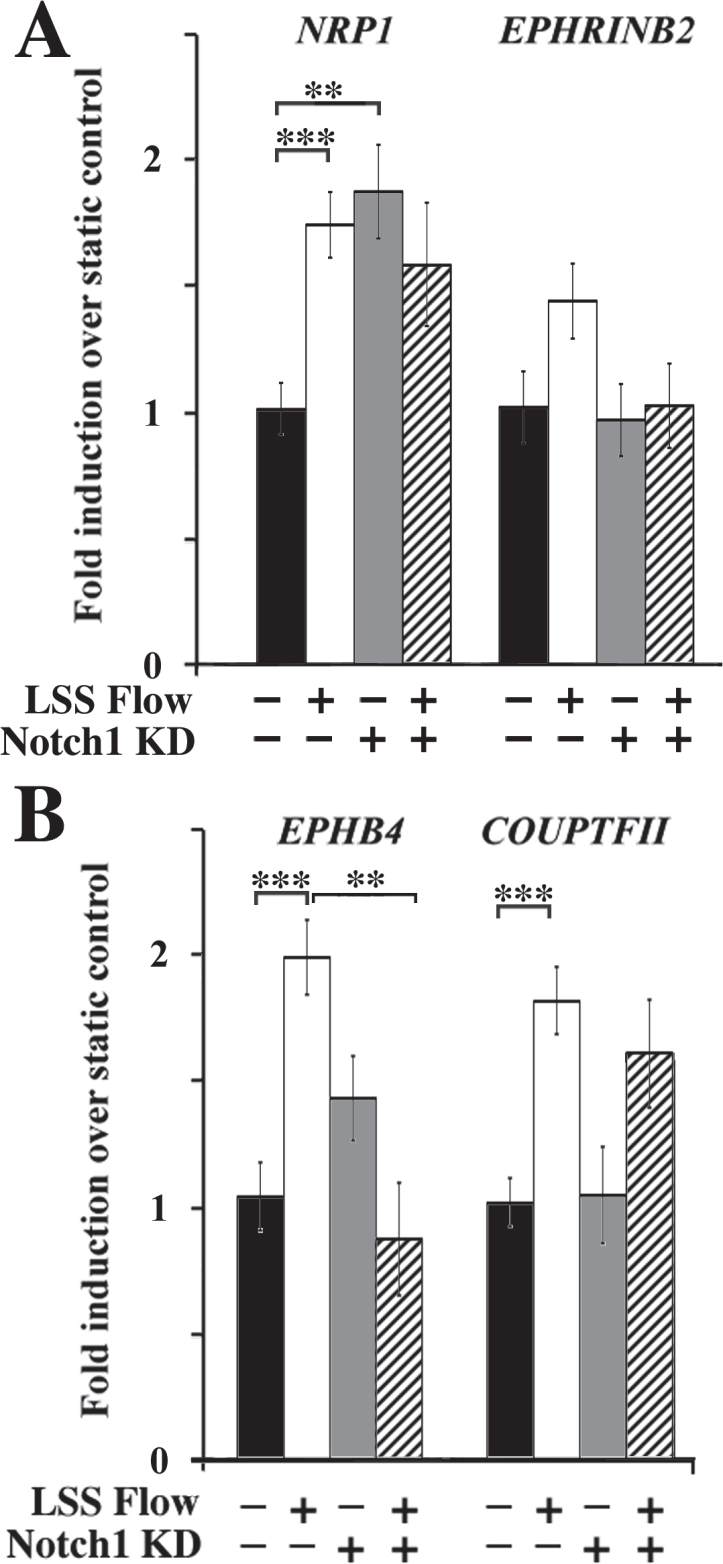
Knockdown of Notch1 does not prevent all shear-induced arteriovenous gene expression. Cells with or without Notch1 knockdown were exposed to low levels of laminar flow (5 dynes/cm^2^) and the expression of arterial (A) or venous genes (B) was examined. All genes investigated were upregulated to a certain extent by laminar shear stress alone (5 dynes/cm^2^), though the change in *EphrinB2* was not statistically significant. Knockdown of Notch1 prevented flow-induced expression of *Nrp1* and *EphB4* but did prevent flow-induced *COUPTFII* upregulation. All values are mean ± SEM (n = 4–16 for all conditions). ** p < 0.01; *** p < 0.001; Two-way ANOVA and Tukey’s post hoc comparisons.

Though endothelial cells have been shown to express some arterial genes before the onset of flow [17], it is also known that the expression of these genes remains plastic, such that an artery will express venous markers if exposed to venous flow and vice versa [5]. We therefore studied whether Notch1 was required for the expression of arterial and venous genes in the presence of flow. Among the genes tested, two arterial genes (*Nrp1*, *EphrinB2*) and two venous genes (*EphB4* and *CoupTFII*) gave the clearest results (Fig 6). Laminar shear stress alone upregulated *Nrp1* (Fig 6A). A modest and not statistically significant upregulation of *EphrinB2* was also observed. We also found that *EphB4* and *CoupTFII* were upregulated by flow. Knocking down Notch1 alone upregulated the expression of *Nrp1*, but blocked further response to shear stress. Upregulation of *EphrinB2* and *EphB4* by flow was also blocked in the absence of Notch1, but not that of *CoupTFII*. Among the genes not shown here, *Nrp2* was affected neither by shear stress nor by Notch1 knockdown. We also tested *Cx40*, which showed a non-significant trend towards increased expression after exposure to shear stress, with and without Notch1 knockdown.

## Discussion

Within the embryo, the cardiovascular system is the first functional organ system to develop. The hemodynamic stresses exerted by the flowing blood on the endothelium play a significant role in regulating many physiological functions as well as inducing changes in gene expression. The Notch signaling pathway is involved in a plethora of biological processes during development and in the adult, including arterial and venous differentiation [15], tip cell formation and regulation [32, 33] and sprouting angiogenesis [32, 34, 35].

In zebrafish, *notch1b and dll4* were identified as arterial-specific genes with the onset of expression beginning prior to the initiation of blood circulation [36]. Contrary to this, our results show that in mouse *Notch1* is expressed in both the dorsal aortae and the anterior cardinal vein before vascular remodeling. Spatial restriction of Notch1 expression cannot be responsible for the induction of arterial identity. Dll4 was recently found to be the earliest expressed arterial marker in mouse embryos [17], and Dll4 preferentially binds to Notch1 [37]. In zebrafish, expression of the Notch1 intracellular domain is sufficient to induce arterial gene expression in veins [16]. If the same is true in mouse, then Notch1 must not be activated in mouse embryonic veins. Dll4, the ligand for Notch1, has been shown to be arterial-specific before the onset of flow [17]. Our results therefore support the hypothesis that the expression of the ligand, rather than the receptor, defines which vessels become arteries. The expression of Notch1 in both arteries and veins also explain why ectopic expression of *Dll4* is sufficient to induce arterialization of the anterior cardinal veins [38].

The early expression of *Notch1* by veins and then the loss of this expression after remodeling is more difficult to understand. It has been suggested that venous identity is a default state of endothelial cells, such that all vessels start out as veins but that Notch activation in a subset of endothelial cells induces those cells to become arterial [39]. The fact that *Notch1* expression is present in venous endothelial cells during early vascular development contradicts this hypothesis. Some signal must be actively turning off *Notch1* expression in the cardinal vein and therefore venous identity cannot be a default state. COUPTFII suppresses Notch1 expression [40]. Our *in vitro* results would suggest that flow in the embryo would upregulate COUPTFII both in arteries and veins. There are differences not only in the level of shear stress but also the acceleration of flow during the cardiac cycle in the early embryo between arteries and veins [3]. These differences may be sufficient to differentially regulate COUPTFII expression such that COUPTFII expression would only be present in veins. This is supported by previous research showing that high levels of steady shear stress induces COUPTFII expression but that pulsatile flow of the same magnitude cannot [41].

We found that flow was required for the expression of Notch1 during development, but that adult artery physiological levels of flow do not induce Notch1 expression. Embryonic shear stress levels can go as high as 5 dynes/cm^2^ during vascular remodeling (between E8.75 and E9.5 [25, 27]), and the velocity of blood flow in arteries is twice as high as in veins [3]. Given that arteries and veins in the early embryo are approximately the same diameter [3], we would expect shear stress levels to be approximately half the magnitude in veins as compared to arteries. Our results show that both 1 dyne/cm^2^ (venous-like magnitude) and 5 dynes/cm^2^ (arterial-like magnitude) induced Notch1 expression to the same extent, explaining why expression is present in both vessels at the onset of flow. Our results also show that the onset of circulation is necessary for this expression to occur. The results are, however, contrary to what has been reported in the zebrafish where ablation of flow resulted in an increase in Notch signaling due to an upregulation of Dll4 [29]. The results in zebrafish also showed, however, that *VEGFR2* (*KDR*) and *ephrinB2* expression are not affected by the ablation of flow and that *VEGFa* expression is inhibited by flow. This is contrary to what is observed in mammalian systems, by us and by many others, where *VEGFR2*, *VEGF* and *EphrinB2* are all upregulated by flow [28, 42–44]. Therefore, it is not clear that results from zebrafish translate to mammals. Furthermore, our results show that the regulation of *Notch1* expression by flow is dependent on the magnitude and type of flow that is present. The levels and patterns of hemodynamic stress present in the zebrafish when circulation begins are likely to be very different than in mammalian embryos.

Our *in vitro* results show that adult artery physiological shear stress levels do not induce Notch1 expression, implying that the continued expression in arteries is not dependent on Notch1 induction by shear stress. Activation of Notch receptors induces a positive feedback loop resulting in the expression of Notch ligands [30, 31]. Similarly, we find that all Notch ligands and effectors can be upregulated by flow, but in the absence of Notch1, flow cannot upregulate these genes. As such, flow can initiate the expression of *Notch1* but sustained signaling from flow may not be required once expression has been initiated.

Notch signaling plays a crucial role in arterial and venous differentiation during development. Although earlier *in vivo* work shows a decrease in Nrp1 expression in the dorsal aorta of *Notch1* knockout mice [24], we observe an increase in *Nrp1* expression with Notch1 knockdown *in vitro*. We achieved a 70% knockdown of *Notch1* in HAAEC, and residual expression of *Notch1* in the endothelial cells might account for this variation. We find that *Nrp1* expression remains unchanged after cells with *Notch1* siRNA are exposed to laminar flow. This indicates that although Nrp1 and VEGFR2 are upstream of Notch1 in the VEGF-signaling pathway [16], there may be a feedback loop in place that alters their regulation under flow.

Though many genes involved in arterial-venous differentiation failed to be upregulated by flow after the knockdown of Notch1, there are exceptions both within the set of arterial specific genes (*Dll1*, *Jag1* and possibly *Cx40*) as well as venous-specific genes (*CoupTFII*), and others (*KLF2*). Hence, our results show that Notch signaling can modulate how flow affects endothelial cells, but it does not appear to be an indispensable protein in the process of mechanotransduction.
